# Supplementary material for: The complete chloroplast genome of Dendrobium nobile, an endangered medicinal orchid from north-east India and its comparison with related Dendrobium species
Source: PeerJ. 2019 Nov 1;7:e7756. doi: 10.7717/peerj.7756 (PMC6830405; doi:10.7717/peerj.7756)
Supplement: Table S1 [file peerj-07-7756-s001.rtf]

Title: fasta fileDescription  Analysis     Analysis ---------------------------- Fisher's Exact Test of Neutrality Selection    Scope ------------------------------- In Sequence Pairs  Substitution Model    Substitutions Type ------------------ Syn-Nonsynonymous    Model/Method ------------------------ Nei-Gojobori method (No. of Differences)  Data Subset to Use    Gaps/Missing Data Treatment --------- Complete deletion  No. of Sites : 36198  Prob (black) : Probability computed (must be <0.05 for hypothesis rejection at 5% level [yellow backgroud])[ 1] #D_nobile_IND[ 2] #D_candidum[ 3] #D_officinale[ 4] #D_fanjingshanense[ 5] #D_xichouense[ 6] #D_wilsonii[ 7] #D_hercoglossum[ 8] #D_moniliforme[ 9] #D_huoshanense[10] #D_falconeri[11] #D_pendulum[12] #D_brymerianum[13] #D_henryi[14] #D_lohohense[15] #D_chrysanthum[16] #D_crepidatum[17] #D_denneanum[18] #D_fimbriatum[19] #D_gratiosissimum[20] #D_wardianum[21] #D_ellipsophyllum[22] #D_aphyllum[23] #D_parishii[24] #D_primulinum[25] #D_devonianum[26] #D_jenkinsii[27] #D_parciflorum[28] #D_spatella[29] #D_exile[30] #D_strongylanthum[31] #D_salaccense[32] #D_loddigesii[33] #D_chrysotoxum[34] #G_schlechtendaliana[35] #G_fumata[36] #G_procera[37] #G_velutina[38] #D_nobile_CH[         1      2      3      4      5      6      7      8      9     10     11     12     13     14     15     16     17     18     19     20     21     22     23     24     25     26     27     28     29     30     31     32     33     34     35     36     37     38 ][ 1]         [ 2]   0.215        [ 3]   0.264  1.000        [ 4]   1.000  1.000  1.000        [ 5]   0.425  1.000  1.000  1.000        [ 6]   0.528  1.000  1.000  1.000  1.000        [ 7]   0.279  0.518  0.518  1.000  1.000  1.000        [ 8]   1.000  1.000  1.000  1.000  1.000  1.000  1.000        [ 9]   0.470  0.490  0.499  1.000  1.000  0.407  1.000  1.000        [10]   0.009  0.028  0.025  0.089  0.053  0.047  0.036  0.137  0.068        [11]   0.018  0.057  0.053  0.133  0.086  0.097  0.081  0.221  0.111  0.485        [12]   1.000  1.000  1.000  1.000  1.000  1.000  1.000  1.000  1.000  1.000  1.000        [13]   1.000  1.000  1.000  1.000  1.000  1.000  1.000  1.000  1.000  1.000  1.000  1.000        [14]   1.000  1.000  1.000  1.000  1.000  1.000  1.000  1.000  1.000  1.000  1.000  1.000  1.000        [15]   1.000  1.000  1.000  1.000  1.000  1.000  1.000  1.000  1.000  1.000  1.000  1.000  0.359  0.143        [16]   1.000  1.000  1.000  1.000  1.000  1.000  1.000  1.000  1.000  0.472  0.428  0.507  0.473  0.160  0.126        [17]   1.000  1.000  1.000  1.000  1.000  1.000  1.000  1.000  1.000  1.000  0.496  1.000  1.000  1.000  0.439  0.421        [18]   1.000  1.000  1.000  1.000  1.000  1.000  1.000  1.000  1.000  1.000  1.000  1.000  1.000  1.000  0.431  0.396  0.234        [19]   0.057  0.120  0.103  0.205  0.119  0.138  0.100  0.176  0.141  0.006  0.012  1.000  0.477  0.327  0.260  0.185  0.210  0.212        [20]   0.212  0.504  0.445  1.000  0.478  0.486  0.393  1.000  0.428  0.133  0.149  1.000  1.000  1.000  1.000  1.000  1.000  1.000  0.005        [21]   1.000  1.000  1.000  1.000  1.000  1.000  1.000  1.000  1.000  0.348  0.394  1.000  1.000  0.313  0.283  0.228  0.485  0.424  0.160  0.499        [22]   1.000  1.000  1.000  1.000  1.000  1.000  1.000  1.000  1.000  1.000  1.000  1.000  1.000  1.000  1.000  1.000  1.000  1.000  1.000  1.000  0.368        [23]   1.000  1.000  1.000  1.000  1.000  1.000  1.000  1.000  1.000  1.000  1.000  1.000  1.000  1.000  1.000  1.000  1.000  1.000  1.000  1.000  0.423  1.000        [24]   1.000  1.000  1.000  1.000  1.000  1.000  1.000  1.000  1.000  1.000  1.000  1.000  1.000  1.000  1.000  1.000  1.000  1.000  1.000  1.000  0.406  1.000  1.000        [25]   0.297  0.499  0.502  1.000  1.000  1.000  0.437  1.000  1.000  0.180  0.172  1.000  1.000  1.000  1.000  1.000  1.000  1.000  0.048  0.367  1.000  1.000  1.000  1.000        [26]   1.000  1.000  1.000  1.000  1.000  1.000  1.000  1.000  1.000  1.000  1.000  1.000  1.000  1.000  1.000  1.000  1.000  1.000  0.469  1.000  1.000  1.000  1.000  1.000  1.000        [27]   0.113  0.128  0.094  0.183  0.127  0.164  0.181  0.207  0.172  0.037  0.035  0.073  0.076  0.033  0.028  0.015  0.038  0.047  0.006  0.037  0.022  0.083  0.078  0.153  0.147  0.134        [28]   0.192  0.280  0.278  0.360  0.236  0.253  0.324  0.382  0.290  0.089  0.074  0.131  0.167  0.066  0.071  0.026  0.086  0.133  0.027  0.120  0.062  0.147  0.105  0.192  0.342  0.271  0.006        [29]   0.343  0.395  0.328  1.000  0.367  0.433  0.427  0.509  0.397  0.127  0.121  0.444  0.306  0.186  0.145  0.086  0.209  0.218  0.048  0.269  0.065  0.221  0.195  0.311  0.479  0.504  0.102  0.194        [30]   1.000  1.000  1.000  1.000  1.000  1.000  1.000  1.000  1.000  1.000  1.000  1.000  1.000  1.000  1.000  1.000  1.000  1.000  1.000  1.000  1.000  1.000  1.000  1.000  1.000  1.000  0.498  1.000  1.000        [31]   1.000  1.000  1.000  1.000  1.000  1.000  1.000  1.000  1.000  1.000  1.000  1.000  1.000  1.000  1.000  1.000  1.000  1.000  1.000  1.000  1.000  1.000  1.000  1.000  1.000  1.000  0.116  0.085  0.468  1.000        [32]   1.000  1.000  1.000  1.000  1.000  1.000  1.000  1.000  1.000  0.391  0.435  1.000  1.000  1.000  1.000  1.000  0.439  0.502  0.379  1.000  0.237  0.447  0.416  0.492  1.000  1.000  0.026  0.050  0.092  1.000  1.000        [33]   1.000  1.000  1.000  1.000  1.000  1.000  1.000  1.000  1.000  1.000  1.000  1.000  1.000  1.000  1.000  1.000  1.000  1.000  1.000  1.000  1.000  1.000  1.000  1.000  1.000  1.000  1.000  1.000  1.000  1.000  1.000  1.000        [34]   1.000  1.000  1.000  1.000  1.000  1.000  1.000  1.000  1.000  1.000  1.000  1.000  1.000  1.000  1.000  1.000  1.000  1.000  1.000  1.000  1.000  1.000  1.000  1.000  1.000  1.000  1.000  1.000  1.000  1.000  1.000  1.000  1.000        [35]   1.000  1.000  1.000  1.000  1.000  1.000  1.000  1.000  1.000  1.000  1.000  1.000  1.000  1.000  1.000  1.000  1.000  1.000  1.000  1.000  1.000  1.000  1.000  1.000  1.000  1.000  1.000  1.000  1.000  1.000  1.000  1.000  1.000  1.000        [36]   1.000  1.000  1.000  1.000  1.000  1.000  1.000  1.000  1.000  1.000  1.000  1.000  1.000  1.000  1.000  1.000  1.000  1.000  1.000  1.000  1.000  1.000  1.000  1.000  1.000  1.000  1.000  1.000  1.000  1.000  1.000  1.000  1.000  0.193  0.193        [37]   1.000  1.000  1.000  1.000  1.000  1.000  1.000  1.000  1.000  1.000  1.000  1.000  1.000  1.000  1.000  1.000  1.000  1.000  1.000  1.000  1.000  1.000  1.000  1.000  1.000  1.000  1.000  1.000  1.000  1.000  1.000  1.000  1.000  1.000  1.000  1.000        [38]   1.000  1.000  1.000  1.000  1.000  1.000  1.000  1.000  1.000  1.000  1.000  1.000  1.000  1.000  1.000  1.000  1.000  1.000  1.000  1.000  1.000  1.000  1.000  1.000  1.000  1.000  1.000  1.000  1.000  1.000  1.000  1.000  1.000  1.000  1.000  1.000  1.000        Table. Results from Fisher's Exact Test of Neutrality for Sequence Pairs 
